# Establishing the cost of Xpert MTB/RIF mobile testing in high-burden peri-mining communities in South Africa

**DOI:** 10.4102/ajlm.v10i1.1229

**Published:** 2021-11-30

**Authors:** Naseem Cassim, Lindi M. Coetzee, Abel L. Makuraj, Wendy S. Stevens, Deborah K. Glencross

**Affiliations:** 1 Department of Molecular Medicine and Haematology, Faculty of Health Sciences, University of the Witwatersrand, Johannesburg, South Africa; 2 National Priority Programme, National Health Laboratory Service, Johannesburg, South Africa

**Keywords:** GeneXpert test, tuberculosis screening, mobile testing, costing

## Abstract

**Background:**

Globally, tuberculosis remains a major cause of mortality, with an estimated 1.3 million deaths per annum. The Xpert MTB/RIF assay is used as the initial diagnostic test in the tuberculosis diagnostic algorithm. To extend the national tuberculosis testing programme in South Africa, mobile units fitted with the GeneXpert equipment were introduced to high-burden peri-mining communities.

**Objective:**

This study sought to assess the cost of mobile testing compared to traditional laboratory-based testing in a peri-mining community setting.

**Methods:**

Actual cost data for mobile and laboratory-based Xpert MTB/RIF testing from 2018 were analysed using a bottom-up ingredients-based approach to establish the annual equivalent cost and the cost per result. Historical cost data were obtained from supplier quotations and the local enterprise resource planning system. Costs were obtained in rand and reported in United States dollars (USD).

**Results:**

The mobile units performed 4866 tests with an overall cost per result of $49.16. Staffing accounted for 30.7% of this cost, while reagents and laboratory equipment accounted for 20.7% and 20.8%. The cost per result of traditional laboratory-based testing was $15.44 US dollars (USD). The cost for identifying a tuberculosis-positive result using mobile testing was $439.58 USD per case, compared to $164.95 USD with laboratory-based testing.

**Conclusion:**

Mobile testing is substantially more expensive than traditional laboratory services but offers benefits for rapid tuberculosis case detection and same-day antiretroviral therapy initiation. Mobile tuberculosis testing should however be reserved for high-burden communities with limited access to laboratory testing where immediate intervention can benefit patient outcomes.

## Introduction

Globally, tuberculosis is one of the top 10 causes of mortality.^1^ In 2017, tuberculosis infected about 10 million individuals and accounted for an estimated 1.3 million deaths among HIV-negative people, with an additional 300 000 deaths among people living with HIV.^1^ The epidemiology of tuberculosis varies widely between countries. In 2017, the tuberculosis incidence in most high-income countries was under 10 tuberculosis cases per 100 000 population compared to between 150 and 400 tuberculosis cases per 100 000 population in most of the top 30 high-burden countries.^1,2^ Countries such as South Africa (567), Mozambique (551) and the Philippines (554) reported over 500 cases per 100 000 population.^1^ As reported by the World Health Organization, there were 227 224 new cases of tuberculosis in South Africa in 2017. Although 322 000 cases of active tuberculosis were diagnosed in 2017 in South Africa, only 65% of the cases were bacteriologically confirmed, with a treatment coverage of 68% (95% confidence interval [CI]: 51–96).^3^

Clinically, a patient is suspected of having tuberculosis based on the following symptoms: persistent cough of 2 weeks or more, persistent cough of any duration for HIV-positive individuals, fever for over 2 weeks, night sweats, and unexplained weight loss (≥ 1.5 kg within 1 month).^4^ Tuberculosis can present with different symptoms and atypical radiologic findings, and the pathological diagnosis has historically been based on acid-fast bacilli smear microscopy and bacteriological culture.^5^ The latter has a higher sensitivity for diagnosing and confirming active tuberculosis than acid-fast bacilli smear microscopy.^5^ The development of polymerase chain reaction tuberculosis assays has improved tuberculosis diagnosis and facilitates early treatment initiation by significantly reducing the time to result to 2 h, compared to 6 months for bacteriological culture.^6^ In South Africa, the Xpert MTB/RIF polymerase chain reaction assay (Cepheid, California, United States) is used routinely for tuberculosis diagnosis using patient sputum. Test results, which determine the therapeutic intervention and management in line with the diagnostic algorithm, are returned within two days.^7^

Tuberculosis incidence rates globally are especially high in the mining sector. In gold mines around the world, an estimated 3000 per 100 000 population are infected.^8^ In South Africa, the mining sector accounted for 7.5% of the national gross domestic product, employing 495 592 workers in 2014.^9^ Mining activities and environments are associated with a high risk of HIV and tuberculosis transmission and the migration of miners to their place of work is known to disrupt tuberculosis detection and care.^10,11^ Given the higher rates of tuberculosis transmission in mines, it is anticipated that the communities where miners live, the so-called peri-mining communities, would also have higher tuberculosis incidence rates.

Due to the higher burden of disease among miners, a framework to address tuberculosis in the mining sector was developed for the Southern African Development Community in 2014.^11^ In March 2015, a comprehensive tuberculosis campaign targeted at inmates in correctional services prisons, mine workers and peri-mining communities was launched in South Africa under the banner ‘Ending SA [*South Africa*] tuberculosis epidemic: Accelerating the response in key populations’.^12^ In response to this call and through the support of the Global Fund, the National Health Laboratory Service and its clinical partner, the Aurum Institute, introduced a funded mobile GeneXpert testing facility to improve tuberculosis diagnosis in peri-mining communities.^13^ This initiative aimed to increase resources to deal with three of the world’s most devastating diseases (HIV and AIDS, tuberculosis and malaria) by focusing on the areas of greatest need.^13^ Mobile testing was targeted at communities with a high burden of disease (high tuberculosis or HIV prevalence) and little or no access to laboratory testing facilities. These included remote areas of the North West and Limpopo provinces in South Africa between 2016 and 2019.^13^ The step-by-step approach to introducing mobile testing included identification of testing needs, execution of a feasibility study, procurement of funding, conducting of the necessary steps and processes to prepare for testing (setup of vehicles and equipment), assay verification, training, competency assessment, identification of measurable outcomes for monitoring, and commencement of testing.

Various studies have demonstrated that mobile testing is feasible, improves access to diagnostics, and may improve linkage to care and decrease time to treatment.^14,15,16,17^ A local study has reported that linkage to tuberculosis treatment was not associated with either sex or service type (mobile versus stand-alone), but older patients were less likely to be linked to tuberculosis treatment.^15^ Mobile testing for HIV, tuberculosis and, more recently, severe acute respiratory syndrome coronavirus 2 can bring diagnostics to where it is needed in high-burden or outbreak communities.^18^ As previously reported in a local study to evaluate mobile versus traditional laboratory CD4 testing, mobile diagnostics could be substantially more expensive.^19^ Mobile testing is not widely used in South Africa, with its use limited to pilot projects or funded studies. However, it should be possible to integrate mobile testing as part of a national tiered laboratory network to extend services^20^ and absorb the higher cost of mobile testing into the national laboratory expenditure allocations.

There is limited local data on the cost to provide mobile Xpert MTB/RIF testing in high-burden communities. Only one local study reported that the cost to detect one tuberculosis case was $1117.00 United States dollars (USD)based on 1385 patients enrolled.^16^ The paucity of local data for mobile tuberculosis testing highlights the need for a comprehensive costing study, which could inform the modalities of providing these services and identify scenarios that are best suited for on-site testing.

The objective of this study was to determine the cost per result and cost per positive result of mobile Xpert MTB/RIF testing and to compare it to the cost of traditional laboratory-based testing.

## Methods

### Ethical considerations

Ethics clearance was obtained from the University of the Witwatersrand (reference number: M160978). Our study did not contain any patient identifiers. No patient consent was required.

### Context

The National Health Laboratory Service implemented mobile testing in three high-tuberculosis-burden districts in South Africa (Kenneth Kaunda, North West, Waterberg, Limpopo, and Sekhukhune, Limpopo). Traditional laboratory-based testing was conducted at the Potchefstroom laboratory, a clinical pathology district laboratory offering a basic repertoire of testing, including tuberculosis testing, in the Kenneth Kaunda district.

### Costing methodology

The costing analysis was undertaken using Microsoft Excel (Redmond, Washington, United States).^21^ A bottom-up costing approach was used to determine the cost per result from a provider perspective; all costs are reported for the National Health Laboratory Service as the provider of mobile tuberculosis testing. All costs (excluding value-added tax) were obtained in South African rand and reported in United States dollars, with an exchange rate of R14.4838 South African rand (ZAR) to the dollar.^22^ The main outcome of interest was the cost per result. The ingredients-based costing approach established annual equivalent costs (AEC) for the following categories of mobile testing: staff (medical technologist and driver), reagents, external quality assurance, vehicle purchase, vehicle operations, laboratory equipment, and coordinator costs to manage testing. For the costing of the traditional laboratory-based Xpert MTB/RIF testing, we reported the following cost categories: staff (medical technologist), reagents, external quality assurance, laboratory equipment, courier logistics, and coordinator costs to manage testing. All laboratory equipment was purchased outright. For traditional laboratory testing, a placement agreement includes the costs for regular maintenance and servicing of the analyser. All data are reported for the 2018 calendar year. The Consolidated Health Economic Evaluation Reporting Standards checklist was used in the preparation of the manuscript.^23^ For laboratory equipment costing, useful life, which refers to the projected lifespan of depreciable equipment, was set at seven years, with a discount rate of 4%.

For the calculation of staff costs, we determined the full-time equivalent hours (the number of hours worked by an employee divided by the number of hours worked by a full-time employee) based on the amount of time employees were assigned to mobile testing and multiplied this by the annual cost to company salary scales to determine the AEC. Reagent and test consumable costs were obtained from quotations received from the Oracle enterprise resource planning system used by the National Health Laboratory Service, and the AEC was determined using annual test volumes.^24^ For external quality assurance, the frequency of panel testing and the number of samples prepared were used to calculate the AEC per site, that is, panels were sent out quarterly, with three samples per instrument. The AECs for vehicle purchase, vehicle operations, laboratory equipment and the coordinator costs were also determined and are described in more detail below. Start-up costs were defined as all AECs associated with the purchase of the mobile vehicle and laboratory equipment. The total cost per result minus the contribution of start-up costs was also determined. We reported the cost per positive result (the cost to find one tuberculosis-positive case) for both mobile and laboratory tuberculosis testing. This was calculated as the AEC divided by the number of tuberculosis-positive results. For mobile testing, it was also possible to use the clinical outcomes data to estimate the diagnostic cost per tuberculosis-positive patient, as well as the cost per patient initiated on treatment (calculated as AEC divided by the number of people that received treatment).

### Mobile Xpert MTB/RIF costing

The costs for the initial start-up of the mobile service were determined and included the costs for the purchase of the vehicles, modifications made to the mobile units (benches, air conditioning), and purchase and placement of equipment on the mobile units. The mobile units were equipped with GeneXpert platform instruments (Cepheid, Sunnyvale, California, United States). This is an automated real-time polymerase chain reaction test for the simultaneous detection of tuberculosis and rifampicin resistance.^25^ Four GeneXpert instruments, as well as one computer per analyser, were placed in each mobile unit for a combined daily testing capacity of 64 samples. Operational vehicle costs were included in the cost per result and comprised maintenance, fuel, repairs, and annual licensing costs. Additional operational costs included costs for procurement of reagents, consumables, specimen collection and quality control materials (internal and external schemes), as well as other miscellaneous costs such as for printing of results. Each mobile testing unit required a driver and a medical technologist. The percentage of time spent offering mobile testing was used for full-time equivalent calculations, ranging from 40% to 80%. The cost to company salary for a coordinator was calculated using historical expenditure data. The AECs for travel, office setup, miscellaneous costs and coordinator costs were also determined (total AEC divided by the number of mobile testing sites).

The test volumes and number of positive results for each mobile unit were reported using bar charts, with the total cost per result presented as a line chart on the secondary *y*-axis. The cost per result without start-up costs and the cost per kilometre were also reported. The number of site visits and kilometres travelled were indicated as text on the charts. For the three mobile units, we reported the correlation between the cost per result and distance travelled.

### Laboratory-based Xpert MTB/RIF comparative costing

As a comparator, the cost per result was determined for traditional laboratory-based Xpert MTB/RIF testing. Initial laboratory setup included the installation of the four GeneXpert systems (Cepheid, Sunnyvale, California, United States) (capacity of 64 samples per day), an air conditioner, a level two biosafety hood and a vortex mixer. Operational costs included costs to procure reagents, consumables, specimen collection materials, quality control materials (internal and external), printer cartridges and paper. The assumptions for these operational costs were similar to those for mobile testing.

The staff complement required to perform mobile testing included a medical technologist and a laboratory manager, who provided minimal supervision. The technologists performed other testing in addition to Xpert MTB/RIF. The costs of the business management unit (coordinator costs) in the North West province were determined and included the following personnel: business manager, secretary, quality assurance coordinator, human resources officers, training staff, and other support staff. To determine the coordinator costs per result, the AEC was divided by the annual test volume for the province. For the courier costs, the annual expenditure for the laboratory was used.

## Results

The three mobile units performed 4866 tuberculosis tests, of which the majority were performed by mobile unit 1 (68.7%). The mobile units covered a total distance of 64 605 km, with mobile units 3 and 1 contributing 73.7% of all travel. A total of 258 healthcare facilities were visited, evenly distributed between the three units. There were 544 tuberculosis-positive samples reported, with an overall tuberculosis positivity of 11.2%. The tuberculosis positivity was 9.6% for mobile unit 1, 16.6% for mobile unit 2, and 10.7% for mobile unit 3. For the period reported, 11 603 tests were done at the Potchefstroom laboratory, of which 1086 were positive (9.4%).

### Mobile testing costs

**FIGURE 1:**
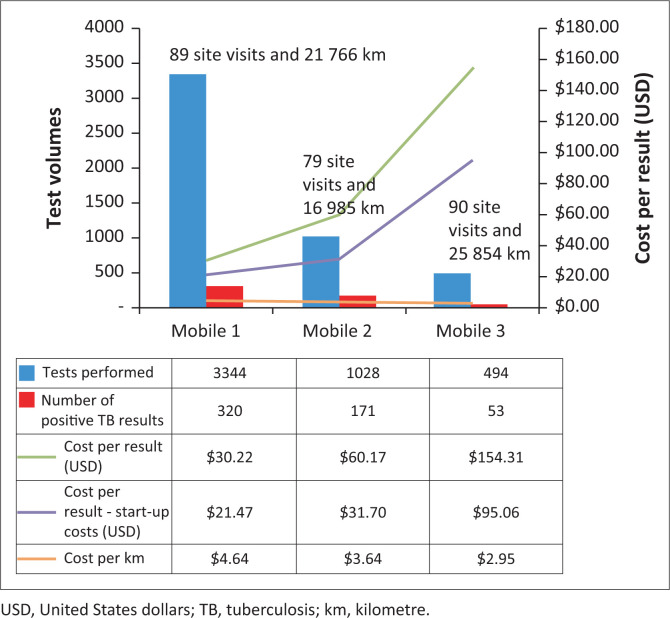
Number of tuberculosis tests performed (dark blue bars) by mobile Xpert MTB/RIF testing units in high-burden peri-mining communities in South Africa, 2018. Positive results (red bars) are reported on the primary *y*-axis. On the secondary *y*-axis, the green line indicates the total cost per result in USD, the purple line indicates the total cost per result less start-up costs, and the orange line indicates the cost per kilometre travelled. The number of site visits for testing and the total distance travelled for those visits are indicated as text for each mobile unit.

The overall cost per result for mobile testing was $49.16 USD with an AEC of $239 130.00 USD (Table 1). Without the start-up costs, the overall cost per result decreased to $31.11 USD. A breakdown of cost contributors showed that staff accounted for 30.7%, primarily due to the cost per result ($11.69 USD; 23.8%) of the medical technologist performing the test. Reagents accounted for 20.7% ($10.16 USD), while vehicle operation costs made up 3.6% ($1.76 USD) of the overall cost per result. Specimen collection and external quality control only contributed 0.5% ($0.27 USD) to the final cost per result. The AEC for reagents, staffing and laboratory equipment made up 72.2% of the total cost. The start-up costs, which comprised the costs to purchase the mobile vehicle and laboratory equipment, accounted for 36.7% ($87 804.00 USD) of the total cost of mobile testing. These initial costs need to be considered when mobile units are rolled out without links to an established laboratory network or testing programme. The cost per result for the three mobile units ranged from $30.22 USD to $154.31 USD. Without the start-up costs, the cost per result ranged from $21.47 USD to $95.06 USD (Figure 1).

**TABLE 1:** Comparison of cost per result between mobile Xpert MTB/RIF testing in high-burden peri-mining communities and traditional laboratory-based Xpert MTB/RIF testing offered at a laboratory in the Kenneth Kaunda district in South Africa, 2018.

Cost category	Mobile testing				Traditional testing			
	Cost per result (USD)	*n*	%	AEC (USD)	Cost per result (USD)	*n*	%	AEC (USD)
Reagents	10.16	-	20.7	49 461.20	10.16	-	65.8	117 940.47
Staffing: Medical technologist	11.69	-	23.8	56 866.12	1.62	-	10.5	18 801.30
Staffing: Driver	3.41	-	6.9	16 600.98	0.00	-	0.0	0.00
Specimen collection materials	0.17	-	0.3	825.01	0.34	-	2.2	3955.03
Test consumables	0.27	-	0.5	1297.16	1.61	-	10.4	18 637.87
External quality assurance	0.10	-	0.2	472.53	0.02	-	0.1	201.40
Vehicle purchase?	7.81	-	15.9	37 992.92	0.00	-	0.0	0.00
Vehicle operation costs	1.76	-	3.6	8540.21	0.00	-	0.0	0.00
Laboratory equipment?	10.24	-	20.8	49 811.96	1.37	-	8.9	15 873.64
Courier costs	0.00	-	0.0	0.00	0.26	-	1.7	2993.39
Coordinator costs	3.55	-	7.2	17 262.69	0.06	-	0.4	728.98
Total cost per result	49.16	-	100.0	239 130.78		15.44	100.0	179 132.08
Less start-up costs	31.11	-	-	-	-	-	-	-
Number of tests performed	-	4866	-	-	-	11 603	-	-
TB+ results	-	544	-	-	-	1086	9.4	-
Cost per result for TB+ results	439.58	-	11.2	-	-	164.95	-	-
On TB treatment	-	300	55.1	-	No data	-	-	-
Cost per result for TB+ patient on treatment	797.10	-	-	-	-	-	-	-

USD, United States dollars; TB+, Xpert MTB/RIF positive; TB, tuberculosis; AEC, annual equivalent cost.

?, Start-up costs.

### Effect of distance travelled on the cost per result

The three mobile units covered distances of 21 766 km, 16 985 km and 25 854 km. The estimated overall cost per kilometre was $2.34 USD, with mobile unit 2 accounting for the highest cost per kilometre ($8.91 USD). The number of health clinics visited by the mobile units ranged from 79 to 90 clinics. The correlation between the cost per result and distance travelled was not statistically significant (*p* = 0.053), with a perfect negative correlation reported (−1.0000).

### Cost per positive tuberculosis result

The AEC for offering mobile testing was $239 130.78 USD to produce 4866 results. There were 544 positive results (11.2%), with 300 patients documented as having received tuberculosis treatment (55.1%). The cost to find one positive tuberculosis case using mobile testing was $439.58 USD and the cost of initiating a positive patient on treatment was $797.10 USD (Table 1).

### Comparative costing analysis

The overall cost per result for laboratory-based Xpert MTB/RIF testing was $15.44 USD (Table 1). Equipment for laboratory testing is procured through a national tender process, that is, there are no costs for installation and maintenance of adequate testing platforms. Reagent costs were similar to that of mobile testing and accounted for 65.8% of the total cost per result. Staff costs contributed $1.62 USD (10.5%) to the cost per result. For specimen collection materials, the cost per result was $0.34 USD (2.2%); for test consumables, the cost was $1.61 USD (10.4%); for external quality assurance, the cost was $0.02 USD (0.1%); for laboratory equipment, the cost was $1.37 USD (8.9%); for the coordinator, the cost was $0.06 USD (0.4%). The courier costs contributed $0.26 USD (1.7%) to the total cost per result.

The AEC for laboratory-based testing was $179 132.08 USD to produce 11 603 results. The cost to find one positive tuberculosis case was $164.95 USD. Unfortunately, the number of patients with a laboratory test result who received tuberculosis treatment was not available.

## Discussion

Mobile diagnostics for high burden diseases such as tuberculosis can provide significant public health and epidemiological value in regions where individuals do not have easy access to laboratory facilities. Overall, the average cost per result for all three mobile units was $49.16 USD. However, the cost per result ranged from $30.22 USD to $154.31 USD, highlighting differences in how and where mobile testing was offered. The biggest contributors to cost differences were test volumes and distance travelled. For example, mobile unit 1 performed the most testing with short travel distances and reported the lowest cost per result. In contrast, mobile unit 3 served a very remote area with longer travel times and had the highest cost per result.

Staff, reagents, laboratory equipment and vehicle purchase contributed 88.1% of the total cost per result. This indicates that the majority of costs associated with mobile testing are not flexible, and suggests that the cost of mobile testing could only be reduced by increasing test volumes, reducing input costs or widening the test repertoire. Test volumes could be increased by identifying clinical settings with higher test volumes that would maximise the use of mobile testing. Test volumes are however limited by the daily throughput of the testing platform and space on the mobile units for multiple units of the test platforms. Negotiations with suppliers could result in lower reagent and consumable pricing. In addition, by adding mobile testing to the existing traditional laboratory national tenders, the placement agreement for reagents and analysers could be extended to mobile testing. The higher test volumes would lower the unit costs of the traditional laboratory supply chain management agreements and, by extension, benefit mobile testing. Various point-of-care platforms with a very small footprint could be used to offer additional routine haematology and chemical pathology mobile testing.^26^ These could be used to facilitate the fast-tracking of antiretroviral therapy for patients with tuberculosis and HIV.^27^

A wide range of tuberculosis positivity rates were reported for the three mobile units in this study. This highlights the importance of identifying high-burden settings with high tuberculosis prevalence for effective deployment of mobile testing. The reported cost to find a single tuberculosis-positive case would vary substantially based on the setting where testing is offered. Offering mobile testing in high-burden areas with a large population would substantially reduce the overall diagnostic cost and simultaneously offer immediate access to treatment. The higher cost of mobile testing should be weighed against the impact of earlier diagnosis, improved coverage, same-day treatment and care, as well as reduced loss to follow-up.^17,28,29,30,31^ Mobile testing as an extension of laboratory testing could also see its higher costs offset by high volume laboratory testing, as bulk testing is still reserved for the laboratory service. The findings of this study confirmed that mobile testing is 3.2 times more expensive than conventional laboratory testing on the same GeneXpert testing platform. Some of the reasons for the higher cost per result for mobile testing include lower test volumes, lost time due to travel to the health facility, and the impact of the clinical workflow on sample collection. An earlier study conducted to determine the cost of providing mobile CD4 testing in Pixley ka Seme in the Northern Cape of South Africa also reported a substantially higher cost for mobile testing versus laboratory testing.^19^ In such remote areas, the cost of mobile testing should be weighed against improving sample collection and distribution routes to the nearest testing laboratory.

For mobile tuberculosis testing, scenarios should be identified that match the increased costs of mobile testing with improved patient outcomes such as rapid tuberculosis case identification and same-day antiretroviral therapy initiation. A clinical outcome study should be embedded within any future mobile testing to assess the impact on patient outcomes. Similarly, detailed cost-effectiveness studies are needed to provide evidence of how mobile tuberculosis testing can save lives and fully realise the potential of targeting high-risk groups.

### Limitations

This study used actual costs from the 2018 calendar year that would be more accurate than a desktop exercise. However, some staffing estimates are based on the typical number of days of mobile testing and this could have underestimated the costs. More so, the costs reported are based on the clinical referral of patients for testing. In a different clinical scenario with higher patient volumes, the costs could be very different. There are several assumptions made for this costing analysis that could have affected the reported cost per result. The number of Xpert platforms in each mobile unit, the level and type of staff employed (technologist versus technician), full-time equivalent assumptions, and the exclusion of some costs, such as overheads, would affect the reported cost per result.

### Conclusion

This study reported that mobile tuberculosis testing is more expensive than traditional laboratory testing. However, mobile testing holds the potential to offer rapid tuberculosis case detection and improve coverage and diagnostics in communities with a high burden of disease. Furthermore, mobiles could be dovetailed to be used to deliver same-day antiretroviral therapy initiation. Further cost-effectiveness studies are needed using the patient outcome data reported.
